# Lenvatinib rechallenge in a patient with advanced thymic carcinoma: A case report

**DOI:** 10.1111/1759-7714.14699

**Published:** 2022-10-17

**Authors:** Yuto Terashima, Taiki Hakozaki, Susumu Takeuchi, Yukio Hosomi

**Affiliations:** ^1^ Department of Thoracic Oncology and Respiratory Medicine Tokyo Metropolitan Cancer and Infectious Diseases Center Komagome Hospital Tokyo Japan; ^2^ Department of Pulmonary Medicine and Oncology Graduate School of Medicine, Nippon Medical School Tokyo Japan; ^3^ Graduate School of Advanced Science and Engineering, Faculty of Science and Engineering Waseda University Tokyo Japan

**Keywords:** lenvatinib, pericardial effusion, pleural effusion, rechallenge, thymic carcinoma

## Abstract

Advanced thymic carcinomas have limited treatment options. Recently, lenvatinib was approved for advanced thymic carcinoma treatment. However, the clinical benefit of lenvatinib re‐administration in patients with advanced thymic carcinoma who developed prior lenvatinib treatment resistance (lenvatinib rechallenge) remains unclear. Here, we present a case treated with lenvatinib rechallenge for advanced thymic carcinoma who was previously treated with lenvatinib as the second‐line treatment followed by multiple cytotoxic agents. Disease control rapidly deteriorated after the eighth line of treatment because of uncontrollable right pleural and pericardial effusion, which required repeated thoracic and pericardial drainage. Shortly after lenvatinib re‐administration, rapid pleural and pericardial effusion reduction was observed. Thereafter, the patient achieved sustained clinical response with good pleural and pericardial effusion control for approximately 7 months. Our case might suggest lenvatinib rechallenge as a treatment option for patients with advanced thymic carcinoma, especially those with poor pleural and pericardial effusion control.

## Graphical abstract

We present a case treated with “lenvatinib rechallenge” for advanced thymic carcinoma who was previously treated with lenvatinib followed by multiple cytotoxic agents. Shortly after lenvatinib rechallenge, rapid pleural and pericardial effusion reduction was observed. Our case might suggest lenvatinib rechallenge as a treatment option for patients with advanced thymic carcinoma.

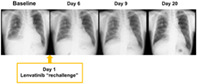

## INTRODUCTION

Thymic carcinoma is a rare and malignant disease,^1^, ^2^, ^3^, ^4^ and the effective pharmacotherapy for advanced thymic carcinoma is heavily limited.^5^ Carboplatin and paclitaxel, or cisplatin, doxorubicin, and cyclophosphamide have often been used as community standard of care for first‐line chemotherapy.^1^ Evidence on the second‐ and later‐line treatment regimens is scant, and cytotoxic agents conformed to advanced lung cancer have been empirically used. Such as S‐1 and gemcitabine.^1^, ^6^, ^7^ The phase 2 National Cancer Center Hospital (NCCH) 1508 (REMORA) trial demonstrated the efficacy and safety of lenvatinib for patients with advanced thymic carcinoma that was previously treated with platinum‐based chemotherapy.^8^ Based on this phase 2 trial, lenvatinib was approved for thymic carcinoma in Japan in 2021. However, a significant paucity of effective second‐ and later‐line treatments for thymic carcinoma remained.

### Case report

**FIGURE 1:**
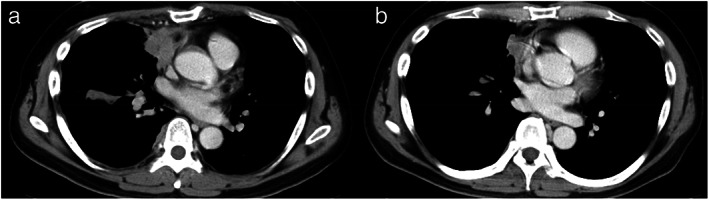
Radiographic results before and after initial lenvatinib. (a) Computed tomography scan showing a primary lesion in the anterior mediastinum before initial lenvatinib. (b) Tumor shrinkage 2 months after initial lenvatinib

A 48‐year‐old Japanese male patient presented with right chest pain and dyspnea. Computed tomography (CT) scan revealed an anterior mediastinal mass, multiple nodular lesions on the right pleura, and right pleural effusion. He was initially diagnosed with thymic carcinoma (squamous cell carcinoma), Masaoka‐Koga stage IVA. His initial Eastern Cooperative Oncology Group performance status was zero. His smoking history was half of one pack of cigarettes for 21 years. Carboplatin and nab‐paclitaxel were administrated in March 2015 as first‐line therapy. A CT scan showed enlarged pleural lesions and increased right pleural effusion after six cycles. The patient was enrolled in the NCCH 1508 (REMORA) trial in October 2017, and lenvatinib was administrated at 24 mg/day as second‐line therapy. He achieved continued response to lenvatinib for approximately 2 years, and the best overall response was the partial response (PR) (Figure 1a,b). During lenvatinib treatment, a gradual lenvatinib dose reduction of 10 mg/day was required because of fatigue and palmar‐plantar erythrodysesthesia syndrome. A CT scan revealed disease progression after 2 years, therefore lenvatinib was discontinued. The patient was subsequently treated with cytotoxic agents, including S‐1, irinotecan, gemcitabine, docetaxel, amrubicin, and vinorelbine, as third‐ to eighth‐line treatment from August 2019 to September 2021.

**FIGURE 2:**
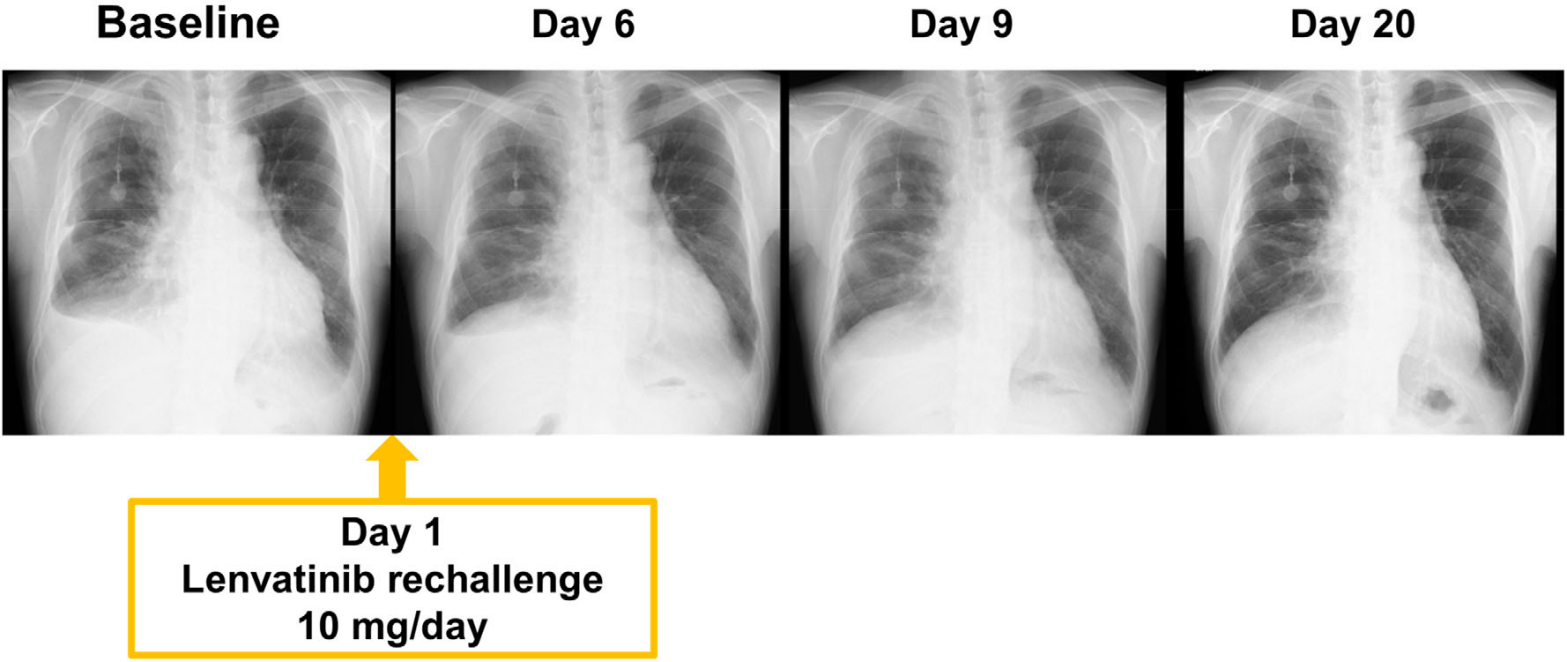
Chest X‐rays before and after lenvatinib rechallenge showing the cardiac size and pleural effusion rapidly decreased after lenvatinib rechallenge

**FIGURE 3:**
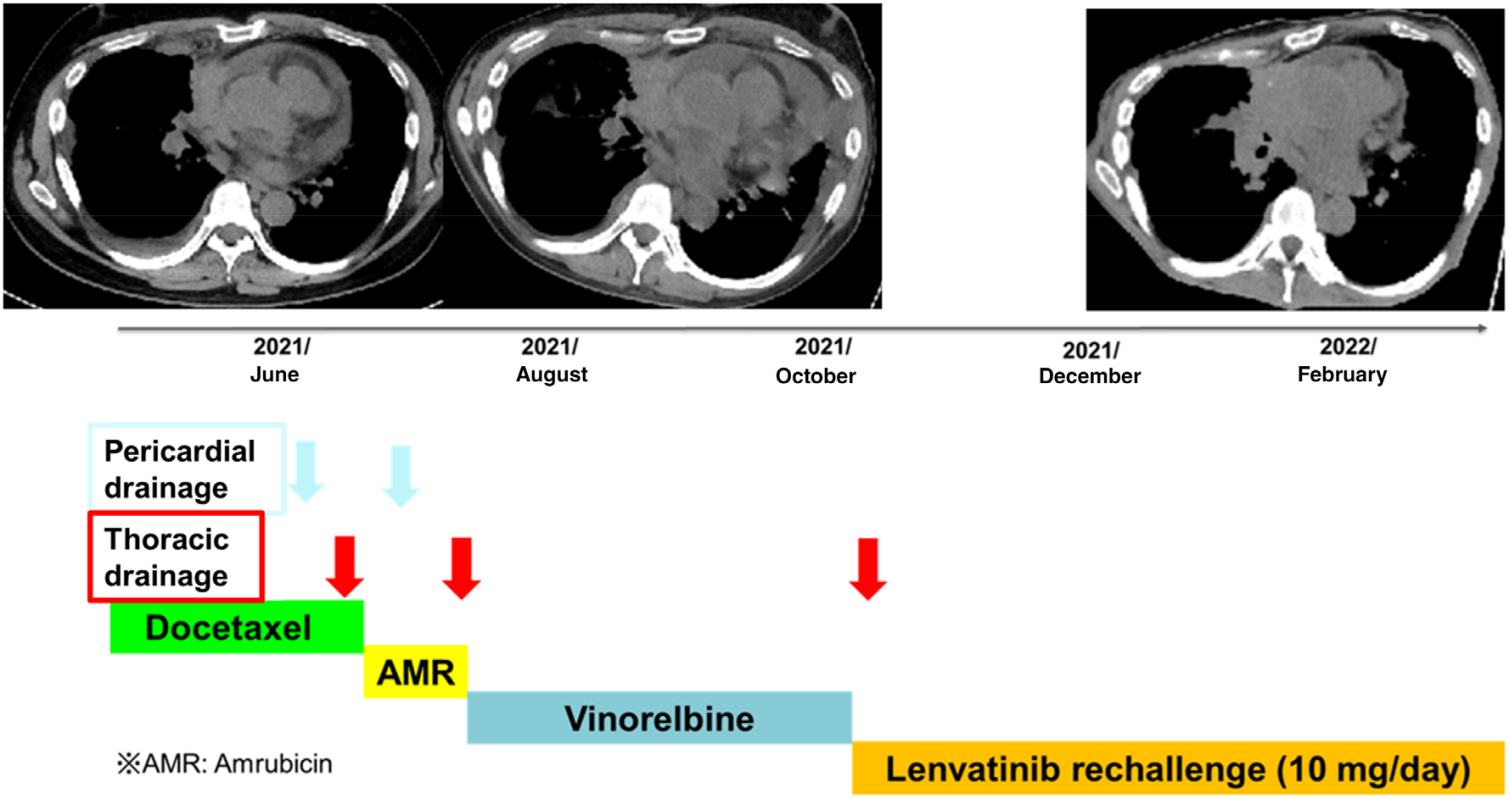
Clinical course and computed tomography (CT) scans before and after lenvatinib rechallenge. The CT scans showed a decreased pericardial effusion and the patient achieved prolonged good disease control after lenvatinib rechallenge

Tumor progression was observed with enlarged anterior mediastinal tumor, liver metastasis, and increased pleural and pericardial effusions despite treatments. Repeated thoracic drainage and pericardial drainage using indwelling catheters were required due to the impending right pleural and pericardial effusion and severe dyspnea. Although we did not perform pleurodesis, we performed intrapericardial instillation of bleomycin following second pericardial drainage. Nevertheless, pericardial effusion was still uncontrollable. Lenvatinib was re‐administrated as ninth‐line therapy. His vital signs at baseline were pulse of 120 beats/min and respiratory rate of 24 breaths/min with an O_2_ saturation of 97% (room air). Lenvatinib was initiated at 10 mg/day, the same as in initial lenvatinib treatment as second‐line therapy. Dyspnea markedly improved and a chest X‐ray showed decreased pleural effusion shortly after lenvatinib rechallenge initiation (Figure 2). The toxicity of lenvatinib rechallenge was acceptable and additional dose reduction was not required. Thereafter, the patient achieved prolonged good disease control for approximately 6 months after lenvatinib rechallenge initiation (Figure 3).

## DISCUSSION

Tumor re‐sensitization after lenvatinib rechallenge was reported in patients with advanced thyroid carcinoma and hepatocellular carcinoma (HCC).^9^, ^10^, ^11^ Two cases of thyroid carcinoma and five of HCC were previously reported (Table 1). Previous reports revealed the relationship between the response to rechallenge with tyrosine kinase inhibitors (TKIs) and the initial response,^12^ as in our study (Table 1). In our case, the initial lenvatinib was administrated for 22.0 months (median duration 8.8 months in the REMORA trial), and the patient achieved PR as the best overall response and sustained the clinical response by lenvatinib rechallenge.^8^ These findings might suggest that lenvatinib rechallenge is effective, especially for patients who demonstrated a clinical response to initial lenvatinib.

**TABLE 1:** Previous reports on cases treated with lenvatinib rechallenge for advanced cancer

No	Author	Year	Sex	Age	Pathological type	Initial lenvatinib			Rechallenge lenvatinib		
						Line	Duration of treatment (months)	Best response	Line	Duration of treatment (months)	Best response
1	Felicetti^10^	2017	F	44	Thyroid carcinoma	3rd	27.0	PR	4th	21.0	PR
2	Takinami^9^	2020	F	72	Thyroid carcinoma	1st	36.0	PR	3rd	NE	PR
3	Komatsu^11^	2021	M	48	Hepatocellular carcinoma	1st	3.1	PR	3rd	2.2	SD
4	Komatsu^11^	2021	M	59	Hepatocellular carcinoma	3rd	4.8	SD	5th	3.2	SD
5	Komatsu^11^	2021	M	66	Hepatocellular carcinoma	1st	10.6	SD	3rd	7.2	SD
6	Komatsu^11^	2021	M	69	Hepatocellular carcinoma	1st	4.4	PR	3rd	12.2	PR
7	Komatsu^11^	2021	M	73	Hepatocellular carcinoma	1st	15.0	PR	3rd	6.8	SD
8	Present case		M	48	Thymic carcinoma	2nd	22.0	PR	9th	6.9	NE

*Abbreviations*: F, female; M, male; NE, not evaluable; PR, partial response; SD, stable disease.

The mechanisms of acquired resistance and re‐sensitization to lenvatinib are not fully understood. Previous reports suggest that both TKI‐sensitive and ‐resistant clones coexist in tumors, and the ratios of these clones change with TKI administration.^9^, ^10^, ^13^, ^14^, ^15^ TKI‐sensitive clones are suppressed with initial TKI treatment, and the patient achieves clinical response. However, the ratio of TKI‐resistant clones gradually increases oversensitive clones with continuous TKI administration, and the tumor becomes resistant to TKI. TKI‐sensitive clones can regrowth in the tumor in the tumor after the next chemotherapy treatment and absence of TKI. TKI rechallenge showed effectiveness against this tumor. In our case, subsequent cytotoxic agent treatment and prolonged lenvatinib interruption may result in a lenvatinib‐sensitive clone predominance.

Lenvatinib is a multitargeted kinase inhibitor for vascular endothelial growth factor receptor (VEGFR), fibroblast growth factor receptor, c‐Kit, and other kinases.^8^ VEGF plays an important role in the development of malignant pleural and pericardial effusions.^16^ Non‐small‐lung carcinoma cells produce and secrete VGEF, and high VEGF levels in serum or pleural effusion induce the development of malignant pleural effusion.^17^, ^18^ Bevacizumab, a VEGF monoclonal antibody that inhibits tumor angiogenesis, is highly effective against malignant pleural effusion. Bevacizumab also plays a predominant role in pericardial effusion treatment by blocking the VEGF–VEGFR signaling pathway and tumor angiogenesis of the serous cavity.^19^ Thus, lenvatinib, which targets the VEGF pathway, is effective even for thymic carcinoma with poorly controlled pleural and pericardial effusions.

In summary, we present a case of thymic carcinoma with refractory pleural and pericardial effusion who demonstrated a drastic clinical response to lenvatinib rechallenge. Our case suggests lenvatinib rechallenge as a treatment option even for patients who experienced disease progression after initial lenvatinib treatment.

## AUTHOR CONTRIBUTIONS

Yuto Terashima: Conceptualization, investigation, validation, visualization, writing – original draft; writing – review & editing. Taiki Hakozaki: Conceptualization; investigation; supervision; writing – review & editing. Susumu Takeuchi: Conceptualization; writing – review & editing. Yukio Hosomi: Supervision; writing – review & editing.

## FUNDING INFORMATION

This report did not receive any specific grant from funding agencies in the public, commercial, or not‐for‐profit sectors.

## CONFLICT OF INTEREST

Yuto Terashima declares no competing interests. Taiki Hakozaki has received personal fees from Chugai Pharmaceutical and Ono Pharma outside the submitted work. Yukio Hosomi has received personal fees from Astra‐Zeneca, Bristol Myers Squibb, Chugai Pharmaceutical, Eisai, Eli Lilly, Kyowakirin, Nippon Kayaku, Novartis Ono Pharmaceutical, Takeda, and Taiho Pharmaceutical outside the submitted work.

## Data Availability

All relevant data are within the manuscript.
